# Formative assessment scores in tutorial sessions correlates with OSCE and progress testing scores in a PBL medical curriculum

**DOI:** 10.1080/10872981.2018.1560862

**Published:** 2019-01-09

**Authors:** Lucélio B. Couto, Marina T. Durand, Amora C. D. Wolff, Carolina B. A. Restini, Milton Faria, Gustavo Salata Romão, Reinaldo B. Bestetti

**Affiliations:** a Department of Medicine, University of Ribeirão Preto, Ribeirão Preto, Brazil; b Pharm & Tox Department, COM-Michigan State University, Lansing, MI, USA

**Keywords:** Formative assessment, medical education, problem-based learning, progress testing, objective structured clinical evaluation

## Abstract

**Background**: Effective assessments programs are a challenge in problem-based learning (PBL). One of the main principles of this educational setting is the Formative Assessment (FA). We hypothesized that students’ performance assessed by FA in tutorial sessions in a PBL curriculum is related to other summative assessments.

**Objective**: To investigate the correlation among FA in tutorial sessions with grades obtained in Objective Structured Clinical Evaluation (OSCE) and Progress Testing (PT) to better understand the assessment process in PBL medical teaching approach and to predict student’s future performance.

**Design**: An observational cross-sectional study was conducted comparing FA, OSCE and PT scores from 4th to 8th semester medical students. Correlation analyses were performed using pooled and separate data from the 4th and 8th semesters.

**Results**: From the 5th to 8th semester, OSCE scores were smaller compared to the FA, while PT scores were lower in all stages. In the pooled data, the correlation analysis showed a significant positive relationship between grades on FA and OSCE, FA and PT and OSCE and PT. A significant correlation among the three assessments strategies was also detected in the 8^th^ semester, but not in the 4th semester.

**Conclusions**: Assessment strategies in PBL approach, including FA, OSCE and PT, have positive correlations, which increases as the medical course becomes more complex.

## Introduction

Problem-based learning (PBL) is an instructional pedagogy in which students play a central role in the construction of their learning. In this setting, several competencies, e.g., knowledge acquisition, practical skills and professional attitudes, are acquired in order that students become active, cooperative and self-directed learners [1]. Accordingly, assessment programs should be consistent and aligned with these PBL curricular tenets [2,3].

The use of effective and reliable strategies to assess the overall performance of students is one of the major challenges in PBL method [4,5]. Most of PBL medical schools use multiple formats of assessments, which provide information on distinct aspects of their competencies [2,6]. In our PBL hybrid curriculum at University of Ribeirão Preto, assessment is summative (SA) as well as formative (FA) [7], especially in tutorial sessions [8]. To do so, we use expert subject tutors [9,10] trained to perform both types of assessment.

Formative assessment (FA) is one of the main principles of educational student-centered settings [2,11], and generally occurs in the context of a clinical problem during the PBL tutorial session [4,11–13]. It evaluates general student’s performance covering multiple expertise, such as students’ preparation, knowledge, ability to integrate concepts, communication, attitude and cooperativity [6,11]. FA gives a feedback to students, informing their present state of learning, assessing factors that are not easily evaluated by objective method, and avoiding negative impact of more formal summative evaluations [11,14]. Hence, FA is a guide to undergraduates about their performance, providing opportunity for them to shape and enhance their competencies [11,15].

Other assessments, for instance Performance-based tests and Progress Testing (PT), are also achieved to compound the summative grade [3]. Performance-based tests, such as Objective Structured Clinical Evaluation (OSCE), is a useful tool for objectively assess clinical reasoning, communication skills, and interpersonal behavior [2,3,6,16–19]. The OSCE consists of a well-planned and structured evaluation of a multiple timed stations focused tasks [3,19]. PT is an approach historically linked to integrated PBL curricula [20–23], and it has been used to testing medical student since the late 1970s [22,24]. It was established to developed longitudinal and comprehensive assessment of students’ knowledge acquisition, since it is periodically given to all the students over the 6-year period of the medical course [25–27].

Considering all different aspects of evaluations, studies have attempted to establish which specific elements in the PBL assessments have a better relationship with academic performance [5]. However, there is scarce literature addressing this issue. Most of studies only investigate the relationship between FA in PBL tutorials and content assessments [5,28–30]. Since OSCE and PT offer to the students a feedback about their performance along the course, which involve not only summative but also formative purposes, we hypothesized that students’ performance assessed by FA in PBL tutorial process correlates with OSCE and PT scores.

Thus, the aim of this study was to investigate the correlation of FA in tutorial sessions with grades obtained from OSCE and PT in an attempt to better understand the assessment process in PBL medical teaching approach and to predict future student’s performance.

## Methods

### The medical course at UNAERP

The medical course of University of Ribeirão Preto follows the Guidelines for Medical Courses of the Ministry of Education and Culture of Brazil. Details of this curriculum have been described elsewhere [7]. Briefly, the medical course lasts 6 years; each semester comprises one stage, which lasts 21 weeks on average. Therefore, in total, the medical course runs 12 stages. The curricular organization seeks interdisciplinarity by integrating activities of theoretical foundation (Tutoring), technical training (Medical Skills) and ability to deal with problems in the community (Primary Care). The contents are presented to the student in a grade of increasing complexity throughout the course.

The first four years encompasses three curricular units, namely: Tutoring, Medical skills, and Primary Care. The last two years are devoted to the Clerkship, where the students rotate in Pediatrics, Gynecology and Obstetrics, Internal Medicine, Family Medicine, Urgency and Emergency, and Surgery.

Tutoring is the curricular unit, which uses the PBL method as a pedagogical tool during the Pre-Clerkship stages. Tutorial modules compose the Tutoring unit. A module is comprised by five problems under the same subject, for example, infectious disease. Each stage runs three different modules, which means that about 15 problems are solved in each stage. Therefore, in total, about 120 problems on average are solved during the Pre-Clerkship period.

Details of Tutoring have been described elsewhere [8]. Briefly, a small group of students meets each week to solve a problem under the guide of a Tutor. This is called Tutorial Session and lasts approximately four hours weekly.

Each Tutorial session is in accordance with the seven jumps of Maastricht [31], and comprises two phases: the analyzing phase and the reporting phase. In the analyzing phase, a problem is showed to students; students try to solve the problem with their current knowledge about the subject under discussion. However, because knowledge is insufficient, they have to establish learning goals to solve the problem with a self-directed study. One week later, in the reporting phase, students meet again and, with the new knowledge acquired, they discuss the problem more in depth to the point of solving it.

During the first four stages of the medical course, the problems encompass the morphophysiological aspects of the human being. From the fifth to the eighth stage, the problems deal with the more prevalent diseases of our region and our country.

All Tutors are teachers at the medical course, which were particularly trained by the staff expert in the PBL method to become a Tutor before guiding a Tutorial session [10]. They are specialized in the subject under discussion in a module, according to students’ requirements, since 2014 [9]. Therefore, a cardiologist runs the module related to heart disease. They are also instructed to elaborate and carry out the different types of assessment. The Tutor function is to probe students to activate previous knowledge on the subject as well as to help the integration of new knowledge in the topic under discussion. In addition, the Tutor helps students to increase intrinsic motivation to solve the problem; by doing that, the Tutor encourages the self-direct study between the analyzing phase and the reporting phase.

The Medical Skills unit is essentially practical. It runs specific laboratories training in simulated environment. The themes developed are aligned to those presented in the tutorial modules, thus seeking the integration between the theoretical and practical teaching. The Medical Skills unit also focuses on performing anticipatory clinical activities with the use of mannequins, electronic devices, demonstrations by teachers, and interpretation by actors. In addition, as the medical course becomes more complex, from the 5^th^ stage onwards, real patients are used in the Medical Skills unit.

The Primary Care Unit focuses on the primary and secondary level of care, and on some aspects of Clinical Epidemiology. Such activities intend to develop students’ qualification for professional practice, with a focus on primary and secondary care, thus enabling them to deal with the most prevalent health problems in our country. The purpose of this unit is to integrate the knowledge obtained in Tutoring and Clinical Skills in order to increase the comprehensiveness of the teaching-learning process.

### The assessment program in the medical course at UNAERP

The assessment program includes FA and SA, which seeks to comprehensively measure the student’s development regarding knowledge acquisition, skills and attitudes during the entire course. From the 1st to the 8th stage, the final score in each module of Tutoring is comprised as follows: 48% by a final written assessment, 12% by the average tests before and after the reporting phase, 10% of the average of FA, and 30% of the average of practical tests. The latter is related to the assessment in the laboratory classes, which illustrates the topics under discussion in the Tutorial Sessions.

In the Medical Skills curricular unit, the grade obtained in the OSCE composes 40% of the final score, whereas the PT grade composes 20% of the final student grade. The remaining 40% of the final grade is comprised by the grades obtained in the practical and theoretical tests got during the curricular unit. The final grade in Primary Care is composed by 60% of a score got in a final written test, 30% by the grade of one activities report, and 10% by a conceptual grade related to participation in such activities. Therefore, neither the PT nor the OSCE contribute to the final student grade in the Primary Care unit. For this reason, the grades got in the Primary Care unit are not used in this investigation.

The assessment in each Tutorial session addresses students’ knowledge and attitudes related to the learning process. Regarding knowledge, the assessment consists of five multiple-choice questions (MCQ), prepared by Tutors, applied before the reporting phase, and another set of five MCQ following the reporting phase. The test applied before the reporting-phase encourages the student to prepare themselves for the tutorial session. The test applied after the reporting phase assesses the knowledge degree obtained and developed during the Tutorial session, according to the learning goals. Immediately after the end of the Tutorial session, the Tutor discusses and correct the tests with students, thus providing feedback for their performance.

With regard to attitude, the Tutor provides a weekly FA to each student about his/her performance in the analyzing and the reporting phases, taking into account pre-specified topics in which the Tutors were previously trained. In this context, attitude is defined as a set of procedures that leads to a certain behavior, which is related to student’s contributions and motivation to take part in the tutorial group discussion [32].

During the analyzing phase, we seek to evaluate students’ motivation for pursuing a strategy to solve the problem, and the ability to apply their previous knowledge about the subject inherent to this problem. We also assess the use of language as an instrument to improve the learning process. In addition, a responsible and respectful attitude towards the whole group and each individual element is evaluated. Thus, in the analyzing phase, FA is related to the ability to solve problems according to:

1. Assiduity and punctuality;
2. Ability to identify the most relevant points of the problem;
3. To use previous knowledge;
4. Ability to formulate, in a clear and concise manner, questions and generate scientifically consistent hypotheses to solve the problem;
5. Positive attitudes towards their colleagues;

Some of the same criteria used in the analyzing phase are also used in the reporting phase, including student’s ability to critically analyze the information brought to the group. Thus, in the reporting phase, students are evaluated according to:

1. Assiduity and punctuality;
2. Previous study related to the proposed themes, being able to bring pertinent information regarding the learning goals previously established;
3. Ability to synthesize and expose knowledge;
4. Critical attitude towards the colleagues for mutual interdependence in the learning process;
5. Positive attitudes towards their colleagues;

These parameters were developed based on the collaborative learning process developed by students, which underlies the PBL methodology, as elaborations, verbalizations, co-construction, mutual support and criticism, and accordance in the cognitive and social domains [1]. In fact, the five criteria take into account the student’s participation in group discussions, the preparedness for the group work by completing independent study, the effective communication during the group work, and the contribution to the group productivity by sharing knowledge to solve the problem [33].

By the end of a tutorial session, when a problem is solved and the subsequent problem is analyzed, the students receive two distinct grades (from 0–10) regarding their participation in each tutorial phase. Tutor considers all parameters outlined above to calculate each grade. For each problem solved, the student receives a score from zero to 10 according to the performance in the analyzing phase; another score from zero to 10 is also given during the reporting phase. The tutor provides feedback regarding the FA grade in a written confidential form to each student at the end of each the tutorial session, allowing the student to be aware of his/her performance. By the end of the module, a final mean score of FA is calculated taking into account all the grades given during each Tutorial session.

In the Tutoring unit, there are also SAs composed by multiple-choice questions (MCQ), before and after each reporting phase [8], and by a final examination at the end of the module consisting of 12 MCQ and four open-ended questions. At the end of each module, a final grade average is calculated based on FAs and SAs to determine whether the student passes or fails the module.

In order to evaluate different skills, the OSCE is applied to students from the 4^th^ to the 12^th^ semesters. The OSCE consists of six active four-minute stations, where student must perform one or more established tasks, such as examining, diagnosing and treating standardized patients, while an examiner evaluates the student’s performance using a scale given in a checklist format. Following a growing scale of complexity throughout the course, the OSCE is able to integrate strategies and knowledge, and is seen as essential for a teaching environment using the PBL method.

The PT is a comprehensive test of the cognitive functions, not linked to any particular curricular unit, covering the entire educational program throughout the six years. However, due to its nature of evaluating the knowledge progress, the PT is directed to the final objectives of the curriculum. This means that the PT assesses the knowledge that the student is supposed to have at the end of the course.

The PT consists of 120 MCQ elaborated with different degrees of difficulty (simple or vignette questions), encompassing the different areas of the medical professional training (Pediatrics, Gynecology and Obstetrics, Internal Medicine, Family Medicine, Urgency and Emergency, Surgery and Basic Sciences). The questions are created to be so comprehensive that it is virtually impossible to study for the PT, thus avoiding students’ to use rote memorization approaches [20,23,27,34].

The PT lasts four hours and is applied simultaneously once per semester to all students in the course, from the first to 12^th^ stage. The individual student’s grade in the PT is calculated considering the number of scores of each student in relation to the scores average of his/her colleagues from the same stage.

### Data collection and statistical analysis

The Scores of FA, OSCE and PT assessments were collected from 312 students from 4th to 8th semesters during July to December/2014. Student had their anonymity preserved. Paired scores were analyzed.

Data are presented as mean ± standard deviation (SD). ANOVA, one-way followed by Newman–Keuls post-test was used to compare the average mean of FA, OSCE and PT grades in the different stages. For correlation analyses between the grades we used Pearson product–moment correlation coefficient followed by test t for correlation coefficient. In order to avoid confounding effect of large samples, we used a Fisher’s large sample z Confidence Interval (FCI), which converts Pearson’s r to Fisher’s z. Therefore, Confidence Intervals of ρ-value were computed using Fisher’s transformation. The steps in computing the FCI for ρ were as follows:

The Fisher *ζ* transformation converted r into the variable ζ which is approximately normal for any value of r as long as n is large enough.

$$\zeta =\;\frac{1}{2}ln\left(\frac{1+r}{1-r}\right)$$

The values of Fisher’s *ζ* in CI were then converted back to Pearson’s r:

(1-α)100% CI for ρ was obtained as follows:

$$\frac{e^{2l}-1}{e^{2l}+1}\leq \rho \leq \frac{e^{2u}-1}{e^{2u}+1}$$

where

$$u=\zeta +Z_{\frac{\alpha }{2}}.\frac{1}{\sqrt{n-3}}$$

$$l=\zeta -Z_{\frac{\alpha }{2}}.\frac{1}{\sqrt{n-3}}$$

GraphPad Prism (5.0) was used for statistical analysis. P-value ≤ 0.05 indicates significant difference.

## Results

From 312 students, 53 were in the 4th semester, 65 in the 5th semester, 65 in the 6th semester, 58 in the 7th semester and 71 in the 8th semester. Table 1 shows the means of FA, OSCE and PT scores. Students from the 5th to the 8th semesters showed lower mean in OSCE compared to FA score. In addition, the PT mean scores in all stages were smaller than the mean of FA and OSCE.

**Table 1.** Means (SD) of Formative Assessment (FA), Objective Structured Clinical Evaluation (OSCE) and Progress Testing (PT) scores of medical school students from the 4th to 8th semesters at UNAERP.

Semester	FA	OSCE	PT
4^th^ (*n* = 53)	7.7 (0.8)	8.1 (0.9)	5.5 (1.3)*#
5^th^ (*n* = 65)	7.7 (0.7)	7.0 (0.8)*	5.0 (1.8)*#
6^th^ (*n* = 65)	7.5 (0.9)	7.0 (0.6)*	5.5 (1.7)*#
7^th^ (*n* = 58)	8.3 (0.4)	7.5 (0.8)*	4.9 (1.7)*#
8^th^ (*n* = 71)	7.4 (1.2)	6.7 (1.1)*	4.7 (1.8)*#

*p < 0.05 compared to FA. #*p* < 0.05 compared to OSCE.

**Figure 1.**
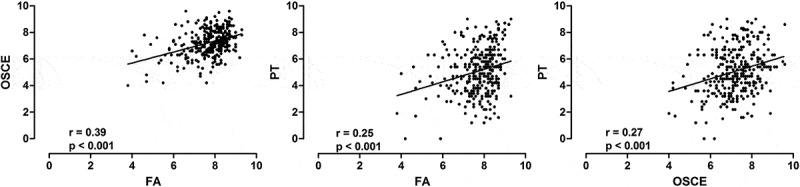
The scores of medical students at UNAERP from the 4th to 8th semesters (pooled data, *n* = 312). Scatter plots of the correlations between Formative Assessment (FA) and Objective Structured Clinical Evaluation (OSCE) (left panel), FA and Progress Testing (PT) (middle panel) and OSCE and PT (right panel). The solid line is the linear regression line. R and p values are shown at the bottom of each graphic.

Despite the fact that there were differences among the averages scores, the pooled data from all stages showed significant positive correlation between FA and OSCE, FA and PT and OSCE and PT (Figure 1). The Pearson’s r, Fisher’s Confidence Intervals (FCI) of ρ-value and p-value are given in Table 2.

**Table 2.** Pearson’s r, Fisher’s Confidence Intervals (FCI) of ρ-value and *p*-value of correlation analyses between Formative Assessment (FA) and Objective Structured Clinical Evaluation (OSCE), FA and Progress Test (PT) and OSCE and PT.

Correlation	Pearson’s r	FCI	*p*-value
**Pool data**			
FA x OSCE	0.39	0.2881 ≤ ρ ≤ 0.4773	<0.001
FA x PT	0.25	0.1461 ≤ ρ ≤ 0.3542	<0.001
OSCE x PT	0.27	0.1656 ≤ ρ ≤ 0.3715	<0.001
***4th semester***			
FA x OSCE	0.25	−0.01990 ≤ ρ ≤ 0.4889	0.069
FA x PT	0.26	−0.009743 ≤ ρ ≤ 0.4966	0.059
OSCE x PT	0.25	−0.02551 ≤ ρ ≤ 0.4846	0.075
***8th semester***			
FA x OSCE	0.48	0.2834 ≤ ρ ≤ 0.6451	<0.001
FA x PT	0.29	0.05866 ≤ ρ ≤ 0.4886	0.015
OSCE x PT	0.32	0.08525 ≤ ρ ≤ 0.5087	0.006

**Figure 2.**
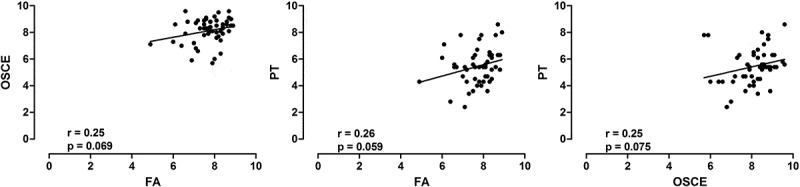
Medical students scores of 4th semester at UNAERP (*n* = 53). Scatter plots of the correlations between Formative Assessment (FA) and Objective Structured Clinical Evaluation (OSCE) (left panel), FA and Progress Testing (PT) (middle panel) and OSCE and PT (right panel). The solid line is the linear regression line. R and p values are shown at the bottom of each graphic.

**Figure 3.**
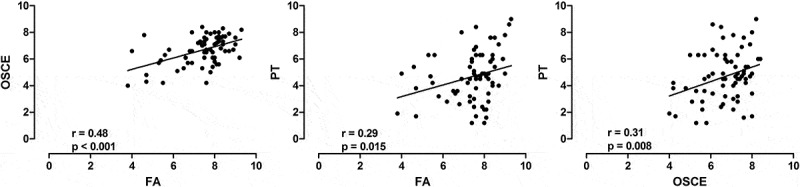
Medical students scores of 8th semester at UNAERP (*n* = 71). Scatter plots of the correlations between Formative Assessment (FA) and Objective Structured Clinical Evaluation (OSCE) (left panel), FA and Progress Test (PT) (middle panel) and OSCE and PT (right panel). The solid line is the linear regression line. R and p values are shown at the bottom of each graphic.

In order to evaluate the students’ progress, we performed separated analysis of the scores from 4th and 8th semester. This is important because the higher the experience of student in the PBL method, the higher the academic performance [8]. In the 4^th^ semester, there is a marginal correlation between FA and OSCE, FA and PT, without statistical significance (Figure 2). In contrast, in the 8th semester, we detected significant correlation among the three assessments strategies (Figure 3).

## Discussion

Over the years, medical schools have endeavored to establish reliable and accurate assessments of students’ competencies. Multiple methods have been applied in an integrated, coherent, and longitudinal fashion [3]. In order to determine the relationship between these methods in PBL curriculum, we performed correlation analysis among the assessments methods used in our institution. Overall, we demonstrate significant correlation between the FA and OSCE and between FA and PT, providing evidence that, during Tutorial session, FA might be an indicator of performance on OSCE and PT. In addition, although OSCE and PT involve evaluation of different competencies, we showed a relationship between them.

Correlations among methods of assessment in PBL are controversial. Von Bergmann et al. [28] observed a significant relationship between facilitator’s assessment of students based on their performance in tutorial groups and content acquisition examination. Yaqinuddin et al. [5] also demonstrated strong association among PBL scores and written examinations, MCQ, short answer questions and OSCE. On the other hand, Kaufman and Hansell [29], and Whitfield and Xie [30] were unable to demonstrate a correlation between facilitators’ ratings and students’ written exam performance. However, both studies present some limitations: non-experts tutors performed the assessments and only students’ knowledge base was evaluated, without considering cognitive and interpersonal skills.

We also showed that grades on FA were higher than those observed in OSCE and PT. This fact may be due to the close relationship between facilitators and students in a long period of contact, which does not exist in OSCE and PT. In fact, there are evidences that tutors tend to over-rate students despite having evidence of poor performance (‘halo effect’) [11,30]. Despite the fact the process of rating students during tutorial sessions has been criticized [6,11,35], current evidences underscore that FA is a reliable indicator of students’ summative learning achievement. Nevertheless, a crucial factor in this process is the qualification of the PBL facilitators [28–30].

In this sense, our group showed that subject-matter expertise among PBL facilitators is essential to all aspects of the PBL learning process [9,10]. Brazilian medical students believe that subject-matter experts perform better for guiding the learning process than their non-expert counterparts [9]. Other authors agree with this concept [5,28,29,35].

A clear comprehension and the proper use of grade criterion by Tutors are also critical during the evaluation of students’ overall performance [5]. It could be one of the reasons why we found the correlations among different types of assessment, such as FA (which assesses attitudes), OSCE (which assesses skills), and PT (which assesses the cognitive domain). Our faculty is trained in PBL processes before joining as a Tutor. They used quite thorough criteria to evaluate the students’ performance during analyzing and reporting phases, which establish a consistent assessment. Having found correlation among FA, PT and OSCE, we assume that these assessments are trustworthy and effective in evaluating students’ cognitive and non-cognitive skills domains. Furthermore, the use of multiple methods of assessments is a manner to obtain a fair judgment of learners.

Assessments of competence play an important role in helping medical students to identify and to respond to their own learning needs, providing insight into their actual performance. In fact, competence acquisition is a developmental process and a habit of lifelong learning, which is nurtured by reflection on experiences [36,37]. Therefore, the assumption that assessment drives learning is one principle of good assessment practice [3,38]. As a result, it is expected that higher levels students might have present better performance in examinations, since their competencies are greater than lower levels. Indeed, we observed a more evident correlation between the scores assessments in 8^th^ semester compared to 4^th^ when analyzed on a semester basis. These data reflect the developmental aspect of students’ competencies, and highlights the importance of metric systems for monitoring student progress in PBL curricula.

In conclusion, the results of the present study show that the assessment process in PBL approach, including FA, OSCE and PT, are significantly correlated. Although the competencies evaluated by these different examinations are not the same and have some degree of subjectivity, the relationship between them is positive and becomes higher during the learning process.
